# The Development and Usability of a Mobile App for Parents of Children with ADHD

**DOI:** 10.3390/children10010164

**Published:** 2023-01-14

**Authors:** Costina-Ruxandra Păsărelu, Reka Kertesz, Anca Dobrean

**Affiliations:** 1 Department of Clinical Psychology and Psychotherapy, Babeș-Bolyai University, 400015 Cluj-Napoca, Romania; 2 The International Institute for the Advanced Studies of Psychotherapy and Applied Mental Health, Babeș-Bolyai University, 400347 Cluj-Napoca, Romania

**Keywords:** ADHD, behavioral parent training, mobile app, parents

## Abstract

Background: Attention-Deficit/ Hyperactivity Disorder (ADHD) is one of the most prevalent mental health problems in children. Behavioral parent training (BPT) is the first-line treatment recommended by clinical guidelines; however, parental dropout is high. Mobile apps could be used as an adjunct to BPT in order to increase treatment adherence, homework compliance, and parental engagement. In this paper, we describe the development process of a mobile app for parents of children with ADHD. Methods: We conducted a study to investigate parents’ perceived usability of the ADHD Coping Card. Results: The mobile app developed has a high usability. Future improvements in the app were suggested by parents. Conclusions: Given the increasing importance of digital mental health interventions in psychotherapy, it is important that future research is conducted with a higher number of participants to investigate the key factors implicated in choosing such an intervention in the future, both by parents and by mental health specialists. A mobile app can be used as an add-on in psychotherapy with parents of children with ADHD. Digital health interventions could help surpass gaps in treatment access for child mental health problems.

## 1. Introduction

Attention-deficit/hyperactivity disorder (ADHD) is a prevalent mental health problem in children, with an estimated worldwide prevalence around 5% [1]. It is affecting multiple domains of functioning, such as personal, social, and academic aspects of life, and there are long-lasting impairments in adulthood as well [2].

However, ADHD not only has a negative effect on the emotional well-being of the child but also affects the family as a whole, with the presence of a child or adolescent with ADHD in the family frequently resulting in increased disturbance in family and marital functioning (e.g., associated with family separation; [3]), affected work efficiency, and a high family burden [4]. A recent meta-analysis indicated a small but significant association between family factors (e.g., parental psychopathology, parenting stress, parenting practices, broken parental partnership) and the severity of ADHD symptoms in children [5]. Higher rates of psychopathology are reported by parents of children with ADHD as compared to parents of children without this diagnosis [6]. Results of a meta-analysis indicate a significant association between child ADHD and maternal depression [7]. Furthermore, parents of children with ADHD display higher levels of psychological distress [8] and a low quality of life [9]. Parental cognitions play a significant role in parental distress; therefore, much research needs to be conducted in order to improve current treatment for ADHD, given that according to previous research, focusing solely on improving the behavior of children with ADHD is unlikely to improve the long-term prognosis [10]. 

Treatment for child ADHD consists of pharmacological, psychological, or combined treatments (both pharmacological and psychological). Pharmacological treatments usually involve stimulant (methylphenidate) or non-stimulant medication administered to children daily, which is effective in reducing core ADHD symptoms such as inattention and distractibility. Psychological treatments encompass cognitive or behavioral interventions [11], such as behavioral therapy, psychoeducation, behavioral parent training, or social skills training. According to clinical guidelines, behavioral parent training (BPT) is the gold standard in the treatment of ADHD in children (NICE guidelines [12]), however, engagement is a major issue that needs to be considered [13]. Homework completion is used as an indicator of treatment adherence, which is positively related to session attendance. Furthermore, it seems that it is a better predictor of treatment response as compared to attendance [14]. Therefore, given all the aspects mentioned, new interventions need to be developed that aim to improve parents’ treatment adherence in BPT. Technology-enhanced BPT [15] could overcome engagement issues, however, limited data exists on such interventions. 

Digital mental health interventions (e.g., delivered through the internet, web or mobile applications, virtual, or augmented reality) are getting more and more popular, especially because of their advantages, which include remote treatment access, reduced costs, the possibility of recording and replaying therapy sessions, or prompt counselor response in crisis situations [16], and they have the potential to overcome barriers in the dissemination of evidence-based interventions. The COVID-19 pandemic showed the potential benefits of digital mental health intervention, given the large adoption of such solutions by mental health specialists [17].

The use of mobile applications as an adjunct to traditional, face-to-face interventions may be effective in augmenting the effects of these treatments. In fact, results coming from a meta-analysis indicate that mobile apps can boost psychotherapy and behavioral interventions’ effects [18], with participants’ who received access to mobile technologies in addition to treatment reporting better outcomes compared to those that do not access mobile apps (moderate effects size, ES = 0.34). While these results are encouraging, research on the use of mobile apps as adjuncts to treatment for ADHD is limited. 

Mental health mobile applications could be cost-effective interventions for children with ADHD and their caregivers [19]. A large proliferation of mobile apps for ADHD in commercially available stores was found in recent years, with numerous mobile apps developed for children diagnosed with ADHD or for their parents that are usually created for evaluating symptomatology, intervention, or both [20]. According to a systematic review of mobile apps developed for ADHD, the science behind the apps available is scarce, with limited research conducted to test their efficacy/effectiveness [20]. 

Several mobile apps have been developed both for children and adults with ADHD, either to improve medication adherence [21], improve classroom behavior [22], or increase physical activity [23]. Results from a randomized controlled trial indicated that a psychoeducational mobile app was superior to brochure-assisted psychoeducation in improving both inattention and impulsivity in adults with ADHD [24]. Furthermore, the same study indicated a higher level of homework compliance in the mobile app group. Another mobile app, delivering psychoeducation for ADHD via a chatbot, was more effective than the information-only control group in reducing ADHD symptoms in adults with attention problems [25]. Given that BPT is the first-line treatment for children with ADHD, we were interested to find mobile apps developed and tested specifically for this population, however, we did not identify any research published on the usability or effectiveness of a mobile app developed for parents of children with ADHD.

It is highly important to involve users in the development of digital mental health information, given that existing apps in commercial app stores may not adequately address parents’ diverse needs [26]. Most of the current mobile applications don’t meet these required characteristics, neither when it comes to scientific criteria (such as using valid assessment instruments or providing evidence-based treatment techniques or information) nor when it comes to users’ specific expectations [20]. Co-designing mobile apps with users can increase engagement, influence their adoption of the technology developed, and increase their satisfaction with the final products [27].

Given the potential advantages of mobile applications in addressing mental health issues, in this paper we present *ADHD Coping Card*, a mobile app for parents of children with ADHD, designed to be used as an adjunct to BPT, aimed to improve parents’ homework adherence and reduce their psychological distress. We investigated the ease of use, parents’ attitudes towards the app developed, and usability of the mobile app. We hypothesized that participants would rate (a) the app as having high usability, (b) the app would be perceived as being user-friendly, and (c) data coming from qualitative assessments conducted with parents would suggest a positive experience with the app. 

## 2. Materials and Methods

### 2.1. Design

A mixed-methods study was conducted with both quantitative and qualitative data collected.

### 2.2. Participants

Participants were recruited online from advertisements posted on Facebook groups for ADHD or for parents. We recruited independent parents; no couples were included. Inclusion criteria: report child has an ADHD diagnosis, have access to a smartphone with an iOS system, speak and write Romanian. We excluded parents who owned a smartphone with an Android operating system (n = 5), since the mobile app was only available in the App Store at the time. The number of participants was determined based on prior usability study research. Four to five participants are required to detect 80% of usability issues [28]. However, we intended to recruit more participants given that, according to other authors, recruiting up to 20 participants is a valid approach in usability testing [29].

Eligible participants were 21 Romanian parents aged between 26 and 45 years old, 19 females and 2 males, with the last education form completed ranging from high school (6 parents), post-high school studies (1 parent), Bachelor’s degree (11 parents) and a Master’s degree (3 parents). Most of the participants’ children in this study were between 2 and 11 years old, out of which 57% were female. All parents declared that they use a smartphone at least four or more times a week. Participants were informed on the privacy, confidentiality, and anonymity of their answers, as well as the fact that they could withdraw at any time from the study.

### 2.3. Description of the ADHD Coping Card App

The ADHD Coping Card App was developed based on an existing BPT protocol for parents of children with ADHD, translated into Romanian [30], that was previously used in a randomized controlled trial where three types of treatment for ADHD were compared (BPT, atomoxetine, or combined treatment) [31]. The contents that address parents’ psychological distress are based on Rational emotive and behavioral therapy [32].

The app is designed to be used as adjunct to BPT and is structured in five sections: a psychoeducation section regarding ADHD, an activities section, a journal section, a mood monitoring section, and a user profile section. 

#### 2.3.1. The Psychoeducation Module

**Figure 1:**
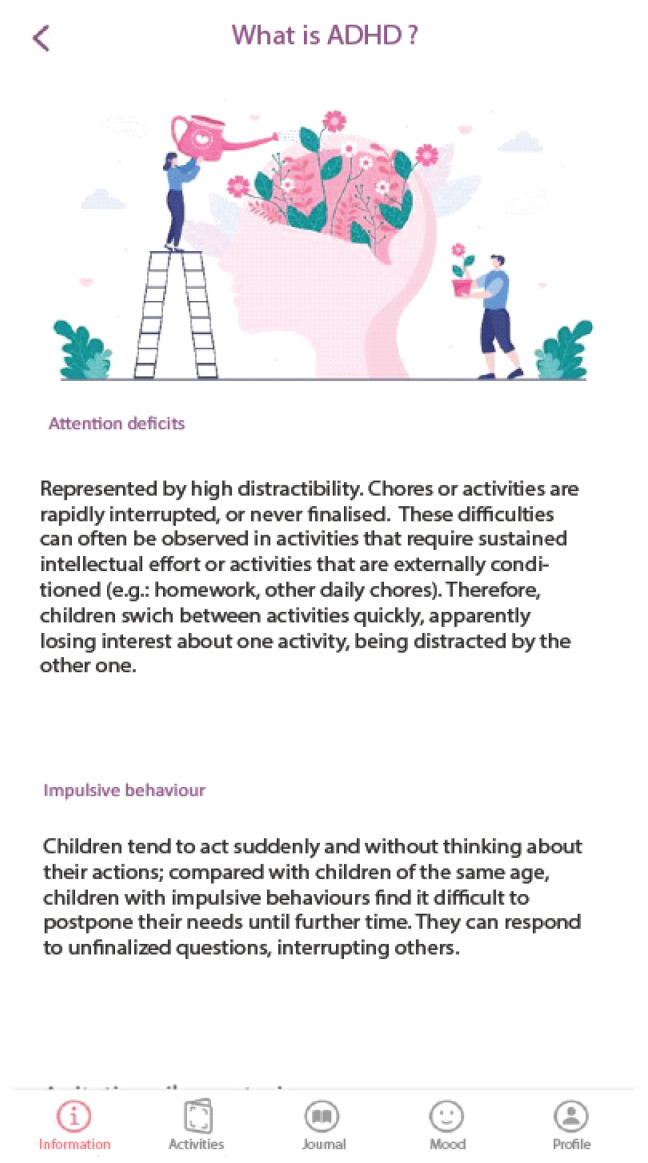
Psychoeducation about ADHD Module.

In this module, general information regarding ADHD symptoms, prevalence, the aetiology of the disorder (the role of genetic and environmental factors), evolution over time, and available treatments is presented (Figure 1). The information is based on evidence-based conclusions about ADHD formulated by the World Federation of ADHD [33]. Psychoeducation is also provided in other modules (see the Journal activities section below). 

#### 2.3.2. The Activities Section

**Figure 2:**
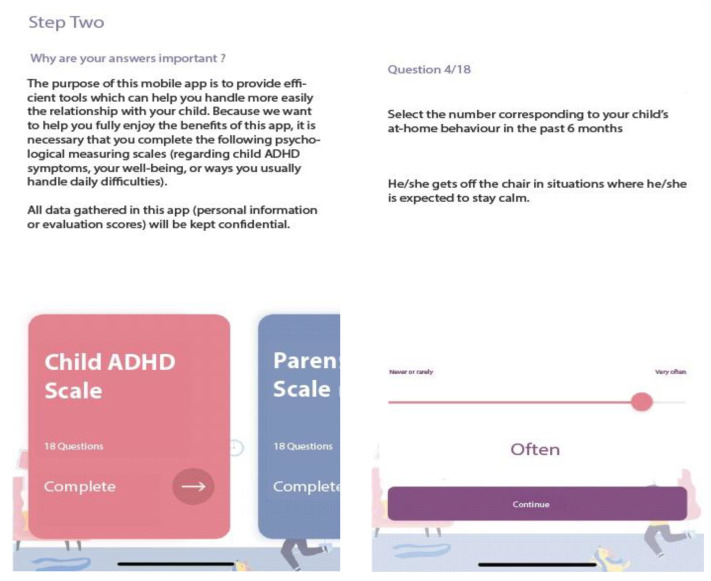
Assessment.

Upon downloading the application, users are required to answer a few questions, such as their age, gender, socio-economic status, residency, family type, child’s age, gender, diagnosis of ADHD, type of ADHD, current treatment, and prior treatment (see Figure 2). 

All this information is stored in the User profile section of the app. Further, several brief instruments with sound psychometric properties, can be completed in the app at the first use and whenever the parent wants to complete each scale (a Progress section is available in Activities) or it is recommended by a mental health specialist to track symptom progress, as follows:

- ADHD symptoms. The Romanian version of the ADHD Rating Scale–IV [34] is used to measure child ADHD symptoms as reported by parents. The scale consists of 18 items, rated on a 4-point Likert scale using never or rarely, sometimes, often, or very often as responses. Scores can be computed for each dimension of ADHD (inattention, hyperactivity/impulsivity). Higher scores indicated higher ADHD symptoms. In the app, the results are presented in percentile scores (derived from the total score), indicating if this could be relevant for further investigation by a mental health specialist. Sample items: “Fails to give close attention to details or makes careless mistakes in schoolwork.”, “Is forgetful in daily activities.”
- Parenting. The Romanian version of the Alabama Parenting Questionnaire Short Form -9 [35] allows the assessment of parenting practices on three subscales: positive parenting, inconsistent discipline and poor supervision. It consists of 9 items (three items for each subscale), rated on a 5-point Likert scale, with responses rated from 1 (Never) to 5 (Always). Sample items: “You let your child know when he/she is doing a good job with something”, “You compliment your child after he/she has done something well”.
- Parental Burden. The Caregiving Burden Scale [36] is used to measure parents’ burden of taking care of children who have physical, emotional, and behavioral problems. It consists of 6 items, each rated on a 4-point Likert scale ranging from 0 (Never) to 3 (Almost everytime), higher total scores indicating more caregiving burden. Sample items: “During the past year, how much worry or concern did [CHILD]’s emotions, behavior, or learning abilities cause you?”, “During the past year, how often have you missed work because of [CHILD]’s emotional, behavioral, or learning problems?”
- Parental distress. The Ultra-Brief Screening Scale for Anxiety and Depression (PHQ-4; [37]) is used to measure parental psychological distress. The scale consists of 4 items (two measuring anxiety, two measuring depressive symptoms), rated on a 4-point Likert scale. PHQ-4 begins with the stem-question, “Over the last two weeks, how often have you been bothered by the following problems?” and responses are rated from 0 (“not at all”) to 3 (“nearly every day”). Sample items: “Feeling nervous, anxious or on edge”, “Little interest or pleasure in doing things”. In this app, we compute a total score on PHQ-4, indicating no-, low-, moderate-, or high-level of psychological distress.
- Satisfaction with the intervention scale. A scale with four questions previously used in another research [38] was used to measure parents’ satisfaction with the mobile app. The scale consists of four questions rated on a 4 point-Likert scale from 0 (Not at all) to 4 (Very much). Sample items: “How satisfied are you with the intervention?”, “Would you recommend this app to a friend?”

**Figure 3:**
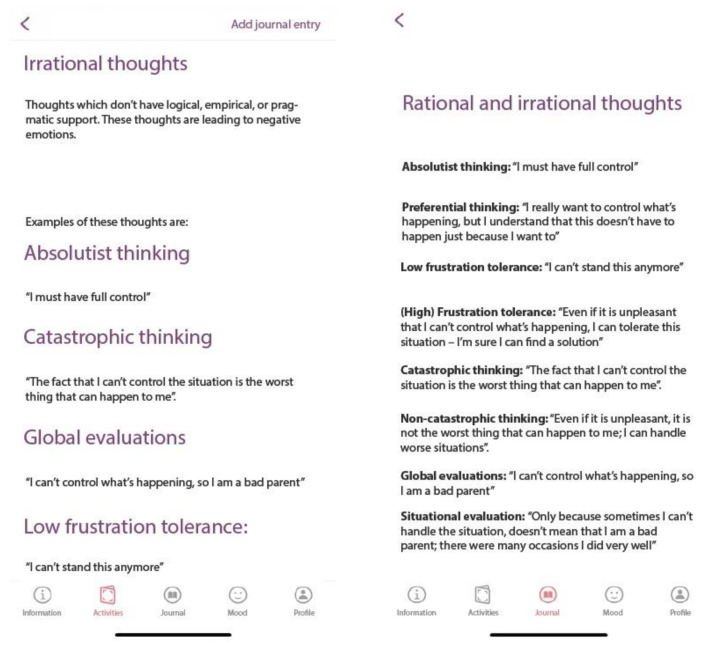
The Activities Section (Irrational beliefs).

The activities section is linked with the journal section (see below). After the presentation of the rationale for each technique or the psychoeducation regarding the ABC model of distress, parents can access the Journal section (see Figure 3).

In order to improve the quality of the relationship between parents and children, two activities are related to the improvement of parent-child relationship, namely one where parents can identify positive aspects of their children and another through which their relationship with their child can be strengthened through play [39]. The Parents guide: observe your child’s qualities is designed to help parents focus their attention on the positive characteristics of their children. The Playing games and fun activities programme is designed to enhance parent-child relationship through engaging in fun activities and playing games. This program focuses on creating a safe space between parents and children where they can have meaningful and playful interactions.

Parental distress section

In this section, the distinction between functional and dysfunctional emotions is made, and the ABC model of distress is presented, where A stands for activating events, B for beliefs, and C for consequences (emotions and behaviors) according to Rational Emotive and Behavioral Therapy (REBT [32]).

Relaxation section

**Figure 4:**
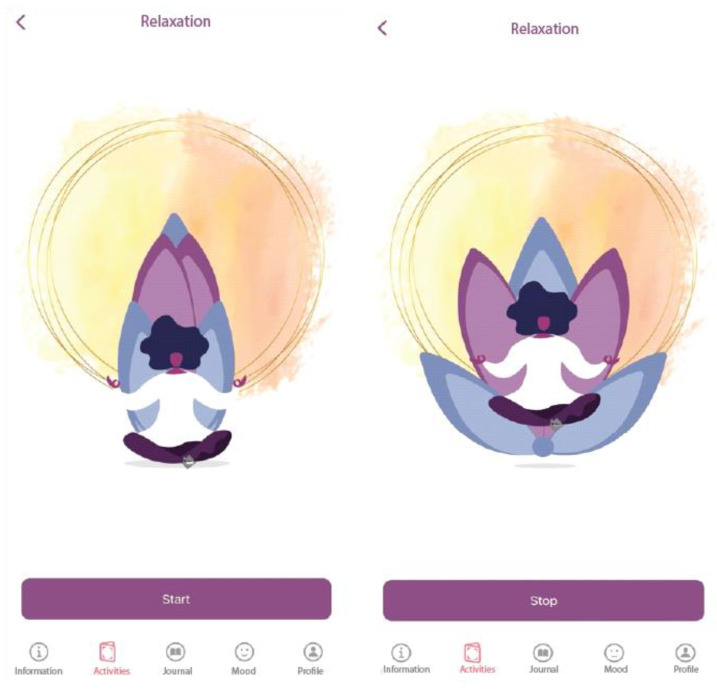
Relaxation in the ADHD Coping Card app.

We implemented a breathing-relaxation section in the app (4-7-8 breathing), consisting of an illustration that expands (3 s) and shrinks (3 s), with a gap of 1 s between the two actions (see Figure 4). Relaxation through rhythmic breathing is proven to help decrease acute/intense (negative) emotions, frequently used in meditation practices as well. Its role in the app is that users can resort to this activity in stressful situations, and they can also use it as a daily practice to help them reduce their physiological arousal. 

#### 2.3.3. Journal Section

Three monitoring journals are available in the app, two related to BPT activities, namely implementing positive parent-child interactions (Parents’ guide of child qualities) and Playing games and fun activities programme, and one related to parental psychological stress (a cognitive restructuring journal). The child’s qualities journal and the fun and play journal, are part of the family activities journal from BPT [30,39] where modalities to focus on the qualities and positive parent-child interactions are promoted. When filling up the journal, parents should select a day from the calendar, write down the activity they participated in on that day, and briefly describe what happened.

**Figure 5:**
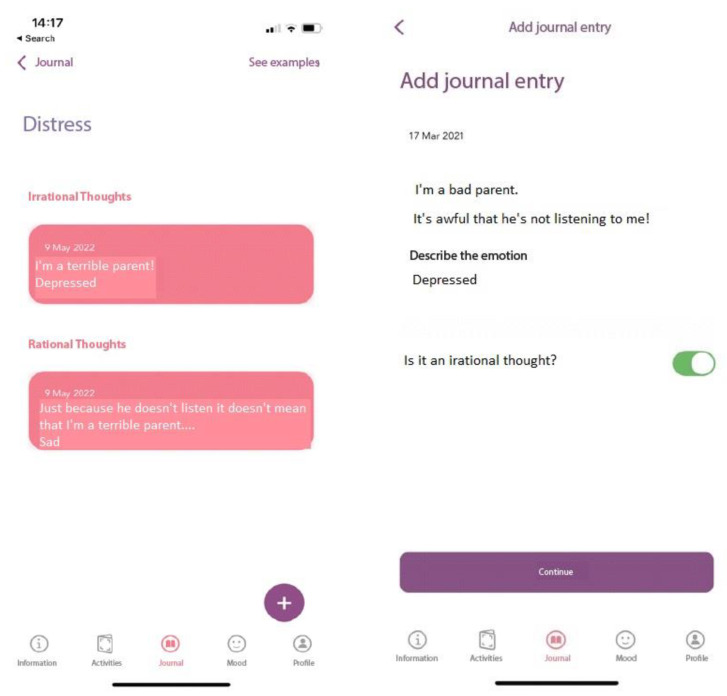
Cognitive Restructuring (Activities linked to Journal).

The cognitive restructuring journal is derived from REBT [32] where irrational beliefs are at the heart of psychological distress. First, an introduction about irrational beliefs is presented, then the four types of irrational beliefs, namely: Demandingness (i.e., inflexible requirements), Awfulizing (catastrophizing), Low Frustration Tolerance, and Global Evaluation (of the self, other people, and/or life) are presented along examples that can be related to parents’ distress. Alternative, rational thinking examples are provided. After receiving psychoeducation about the ABC model of distress and the distinction between rational and irrational beliefs, app users can monitor their thinking patterns and try to identify and change their irrational thoughts using the *Journal* linked to this section (see Figure 5). As we aim through this app is to reduce parental psychological distress and to increase parental engagement with BPT, we included the cognitive restructuring section to assist parents in adopting a more flexible thinking regarding parenting children with ADHD, and their role in caring for them.

#### 2.3.4. The Mood Monitoring Section

**Figure 6:**
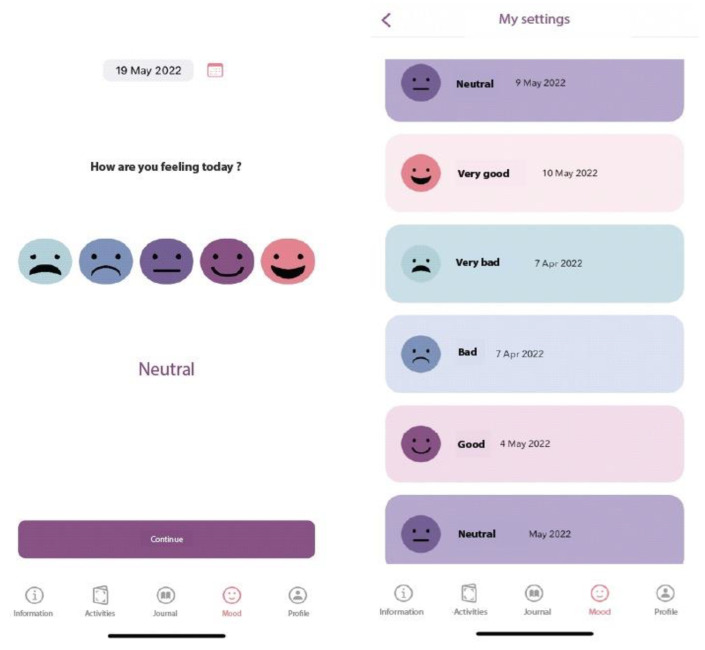
Mood Monitoring in the ADHD Coping Card app.

This can be used independently for daily monitoring of one’s mood, or can be linked to the cognitive restructuring journal, allowing the user to track if his/her emotional state changes over time after learning to dispute and replace irrational beliefs with rational alternatives (see Figure 6). Parents choose a date from the calendar and select an emoji that reflects their mood. A review of mood monitoring is presented, and a complete rating history is made available to the user. 

### 2.4. Measures

#### 2.4.1. Demographic Information

Parents completed a demographic questionnaire regarding their age, gender, children’s age and gender, educational level. 

#### 2.4.2. Technology Use

General information about technology use, such as how often they use a smartphone, key aspects of a mobile app that parents consider to be important, and if they already use online educational or parenting platforms, either ADHD-specific or not.

#### 2.4.3. Parents’ Attitudes Regarding Online Platforms

Parents were asked how useful they find digital mental health solutions addressed to parents of children with ADHD. Answers are rated on a 3-point Likert scale, ranging from 1 (*useful*) to 3 (*neutral*).

#### 2.4.4. App Usability

We used the System Usability Scale (SUS; [40,41]). It provides a global measure of system satisfaction, used across a wide range of user interfaces, such as standard OS-based interfaces, Web pages, Web applications, cell phones, landline phones, and others. The scale contains 10 items referring to customers’ or users’ satisfaction with the used product. Items are rated on a 5-point Likert scale, from 1 (*Strongly disagree*) to 5 (*Strongly Agree*). Scores range between 0 and 100, with higher scores indicating higher usability of the platform. Some items of this scale are: “I think that I would like to use this system frequently”, “I think that I would need the support of a technical person to be able to use this system”, “I need to learn a lot of things before I could get going with this system”. Cronbach’s Alpha was good for the total scale (=0.78).

#### 2.4.5. User-Friendliness

Parents were asked “How friendly is the ADHD coping card app?” with ratings on a Likert scale, ranging from 1 (*Not at all*) to 9 (*Extremely friendly*).

#### 2.4.6. Qualitative Feedback

First, parents were asked which characteristics should have a mobile app in order to be considered useful by parents of children with ADHD. Next, they were asked what they liked and disliked about the mobile app, as well as which features they would delete or add. Next, parents were asked to recommend several improvements in the app, and finally, to indicate any barriers to using the ADHD Coping Card app.

### 2.5. Procedure

**Figure 7:**
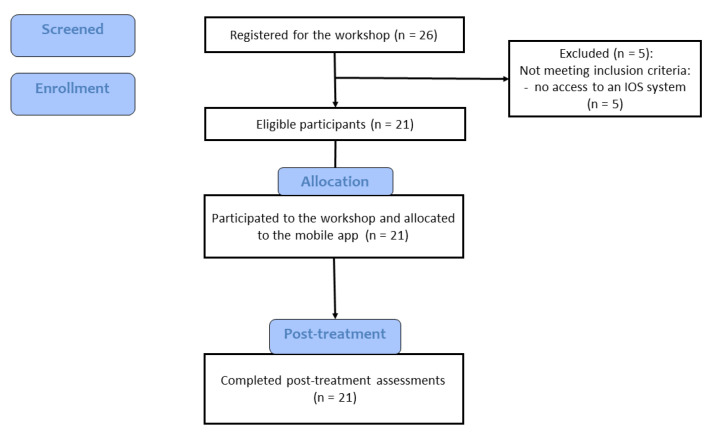
Flow chart of participants through the study.

The study was approved by the Ethics Committee of Babeș-Bolyai University (approval no. 3772/08.04.2022). Participants were recruited online, from Facebook groups for people with ADHD, or for parents of children with ADHD. Interested parents signed the informed consent form and participated in an online workshop with a research assistant. The duration of the workshop was around 45 minutes. The ADHD Coping Card app was presented on a screen, and the content of the modules and available functions were briefly described. After the workshop ended, parents completed an online questionnaire regarding the usability of the ADHD Coping Card and provided feedback on the mobile application (Figure 7).

### 2.6. Statistical Analysis

Qualitative data was analyzed with thematic analysis. Quantitative data were analyzed using SPSS version 26 (IBM Corp). We also conducted two non-parametric Mann-Whitney U tests to investigate if there is a significant difference in the app’s perceived usability depending on parents’ age and parents’ educational level.

## 3. Results

### 3.1. Parents’ Attitudes Regarding the Use of Digital Mental Health Applications

When asked about the perceived usefulness of digital mental health solutions, 57.1% of the parents participating in this study said that digital solutions would be useful tools in helping them with mental health struggles, with 23.8% having a neutral opinion and another 19% considering them very useful.

### 3.2. ADHD Coping Card Usability as Rated by Parents

The usability of the mobile app was good (*M* = 80.35, *SD* = 11.16) which indicates an adequate product [42]. For conducting the Mann-Whitney U test to investigate SUS score differences depending on parents’ educational level, we created two groups: low education level (meaning parents who have high school diplomas, n = 7) and high education level (parents who have bachelor’s or master’s degrees, n = 14). We explored whether parents with a higher educational level will rate higher apps’ usability. Results indicated that the Mann-Whitney test is non-significant for the SUS scores (Asymp.Sig. 2-tailed = 0.312; Exact Sig = 0.322). This means that SUS scores in the low education level group (Mdn = 28) were not significantly different from SUS scores of parents with higher educational level (Mdn = 27.5), U = 35.5, z = −1.01. 

For conducting the Mann-Whitney U test to investigate SUS score differences depending on parents’ age, we created two groups: under and including 31-year-old (n = 11), and over 31 (n = 10) based on the median from our sample of parents. We explored whether younger parents are more likely to be interested in using such an app and will consider it more beneficial than older parents. Results on the Mann-Whitney U test when investigating SUS scores depending on parents’ age are also non-significant (Asymp.Sig. 2-tailed = 1.000; Exact Sig. = 1.000). This means that SUS scores in the under and including 31-year-old group (Mdn = 27) were not significantly different from SUS scores of parents over 31 (Mdn = 28), U = 55.0, z = 0.000. 

### 3.3. User Friendly

The app was rated as friendly, M = 8.33, SD = 1.46.

### 3.4. Qualitative Feedback Regarding App and Future Improvements

In Table 1 parents’ responses to open ended questions were grouped on themes. 

The first aspect mentioned by parents refers to the log-in procedure of the app. Currently, to log in to the app, parents complete a set of demographic data and psychological assessments which are mandatory. Parents’ feedback suggests that this mandatory data completion can be an obstacle to using the app in the future; especially parents of young children perceive a time limitation in their daily lives, so if they do not have time to complete those fields at the beginning, it is less likely that they will do it later. A possible remediation of this aspect could be to make those psychological assessments optional and fillable at any time, so parents can have access to the information and activities in the app even if the questionnaires are not yet completed. This could lead to a greater and better user experience for parents, considering that the scope is to manage their difficulties and not help them complete homework assignments. 

Another important feedback we received is about the way psychoeducation, activities, and information are provided. Currently, the majority of the information is presented in text format, requiring parents to read each activity, module, or psychoeducational feature. This also demands much time and effort for parents to read, understand, and retain. Most parents found it exhausting to read all the information provided in the app, though they appreciated the content, and found it generally interesting and helpful for their circumstances. This feedback could be addressed by integrating into the app more dynamic features that deliver the same information, such as animated videos with voiceover, internet links to verified content about the domain of interest, or other interactive activities built into the app, such as a chat or forum where parents can talk to each other about their challenges. 

The third aspect mentioned by parents was related to the topics presented. Most parents said that they would appreciate information and modules about other possible psychological problems that can be related to child ADHD, such as oppositional defiant disorder, conduct disorder, separation anxiety, or learning disorders, given the high co-occurrence of these disorders in children diagnosed with ADHD. In addition, they suggested the incorporation of additional information or even more modules pertaining to parental distress, anxiety, or depression, so as to have a comprehensive approach toward child ADHD and parental difficulties linked with it. Introducing new modules on a variety of psychological topics might facilitate the dissemination of more valuable information on mental health in general.

## 4. Discussion

In this paper, we describe the development and usability of the ADHD Coping Card, a mobile application aimed to be used as an add-on to traditional, face-to-face, BPT in order to increase parents’ adherence to homework in BPT and to reduce parental distress. Given the promising results of using digital mental health interventions to promote child and parent mental health [43] and the limited research conducted in order to develop mobile applications for ADHD [20], the goals of this study were to present the app’s development with users, investigate its usability, and integrate feedback regarding the app developed to address parents’ needs.

The mobile app developed is complex, aiming to provide both assessment and treatment techniques. Assessments provided in the app are based on instruments with adequate psychometric properties that were previously validated with Romanian samples. The techniques used in the mobile app (psychoeducation, strategies to enhance the parent-child relationship, and cognitive restructuring) are derived from evidence-based interventions for child ADHD [30,39] and for parental distress [32]. Our findings indicated that parents rated the app as easy to use and friendly. The usability of the app was high, and feedback regarding future improvements was provided. When comparing SUS scores depending on parents’ educational level and age, there were no significant differences that could explain which factors contributed more to the perceived usability of a mental health mobile app.

Most parents who subsequently participated in this study showed interest in using mobile applications both for themselves and for their children and considered ADHD Coping Card to be potentially useful for parents. Amongst the key features identified by parents as important in selecting such an app were: a user friendly interface with a transparent and easy-to-use navigation system, evidence-based content about a specific disorder (in this case, ADHD), written in a non-scientific language so it can be easily understood, activities that can consolidate parent-child relationship. The main functionality that parents recommended eliminating from this app was the mandatory psychological assessments from the first stage of creating the user profile in the app. Given that a high percentage of parents of children with ADHD have themselves this diagnosis [44] and that parental ADHD is related to poor effects of BPT [45,46], we need to consider other information delivery modalities through the app instead of text contents, that are more engaging and easier to follow. Furthermore, the dosage of techniques in the app needs to be carefully considered given that according to a meta-analysis, more psychoeducation has a negative effect on parental outcomes, while more techniques involving the manipulation of antecedents of behavior and reinforcement of desirable behaviors are related to parental mental health and parenting [47].

### Limitations and Future Research Directions

This study has several limitations worthy of mentioning. First, the characteristics of the sample are important to take into consideration in order to limit generalizations to other samples. As most of the participants were mothers, all from the same country and mostly living in the same region, we were unable to evaluate how parents from other regions and with different cultural, economic, or educational backgrounds would rate the design and characteristics of the developed mobile app. In addition, since we recruited participants online, our sample may be typical of parents with advanced digital skills and treatment-seeking intentions but not of other parents of children with ADHD.

Second, since we did not conduct interviews to establish a formal diagnosis, parents that participated in this study may have children at risk of ADHD or without a diagnosis of ADHD. Future research could involve parents of children diagnosed with ADHD, as well as mental health professionals who could use the app in their practice.

Another important limitation is related to the small sample size. Our study was underpowered to detect possible differences between age categories or educational levels; therefore, further research should consider this aspect when investigating parental characteristics that can influence technology adoption. The app is available only on one commercial store, therefore, developing an Android version of the app could target a larger population. 

As the objective of the present study was not to validate the digital assessment in ADHD, future research needs to establish the psychometric properties of the app-based assessment and validate it.

## 5. Conclusions

The scope of this study was to present the development of a mobile application designed to assist parents of children with ADHD through BPT and to investigate its usability. Participants were parents of children with ADHD symptoms who attended a workshop where the novel mobile app developed was presented and they interacted with the app. Parents rated the app as having high usability and being user-friendly. Qualitative feedback was obtained to further develop the mobile app. Overall, parents’ ratings were positive regarding the apps’ design, parent-child relationship activities section, and the information provided. Recommendations regarding several features of the app, such as making the assessment module optional or delivering information in a different format (e.g., audio, or video), were also provided by parents in order to further develop the mobile app. Given the increasing importance of digital mental health interventions in psychotherapy, it is important that future research is conducted with a larger sample size to investigate the key factors that will influence the future selection of such an intervention by both parents and mental health professionals. Our study indicated that a mobile app for parents of children with ADHD is a promising approach that could be used as an adjunct to traditional evidence-based treatments.

## Figures and Tables

**Table 1:** Descriptive data about parents’ feedback on ADHD Coping card.

	Theme 1	Theme 2	Theme 3	Theme 4	Theme 5	Theme 6	Theme 7
1. What characteristics should a mobile app have in order to be useful to parents?	Simple information (3 parents)	Relevant and valid information (10 parents)	User friendly (5 parents)	To be able to use it with children (2 parents)			
2. What did you liked about this mobile application?	The design (8 parents)	User friendly and simple information (9 parents)	Activities that target parent-child relationship (8 parents)	Relaxation module (6 parents)	Mood monitoring module (6 parents)	The journals (3 parents)	Parental distress module (3 parents)
3. What didn’t you like about this mobile application?	The delivery form of the information (7 parents)	Psychological assessments (3 parents)	The difficulty level of the information (2 parents)	The small number of parent-child activities (2 parents)			
4. Which functionalities would you eliminate from the app?	Psychological assessments (6 parents)	Psychoeducation module (1 parent)	The relaxation module (2 parents)				
5. What functionalities would you add to the app?	Information about other psychological difficulties (5 parents)	Video and audio recordings rather than text information (4 parents)	More parent-child activities (3 parents)				
6. App improvement suggestions	A simpler way to deliver the content (6 parents)	To remove mandatory activities (1 parent)	To replace the text content with video/ audio recording (2 parents)				
7. Barriers identified for using of this mobile app	Age (1 parent)	Time resources (6 parents)	Financial barriers (3 parents)	Technical knowledge (1 parent)	Parents’ patience (3 parents)		

## Data Availability

The datasets generated during and/or analysed during the current study are available from the corresponding author on reasonable request.
